# Humoral immunity trends in a hemodialysis cohort following SARS‐CoV‐2 mRNA booster: A cohort study

**DOI:** 10.1002/hsr2.1858

**Published:** 2024-02-13

**Authors:** Eibhlin Goggins, Binu Sharma, Jennie Z. Ma, Jitendra Gautam, Brendan Bowman

**Affiliations:** ^1^ Division of Nephrology University of Virginia School of Medicine Charlottesville Virginia USA; ^2^ Public Health Sciences University of Virginia School of Medicine Charlottesville Virginia USA

**Keywords:** booster, COVID‐19 vaccination, end stage kidney disease, hemodialysis, humoral immunity, SARS‐CoV‐2

## Abstract

**Background and Aims:**

Patients with end stage kidney disease on hemodialysis are vulnerable to SARS‐CoV‐2 infection. Current guidelines recommend boosters of SARS‐CoV‐2 mRNA‐based vaccines. The long‐term humoral response of hemodialysis patients infected with SARS‐CoV‐2 after receiving a booster of SARS‐CoV‐2 mRNA‐based vaccines has been incompletely characterized. Here, we determined the long‐term humoral response of hemodialysis patients to two and three doses of the Pfizer BioNTech (BNT162b2) mRNA SARS‐CoV‐2 vaccine and investigated the effect of postbooster SARS‐CoV‐2 infection on antibody levels over time.

**Methods:**

Samples were collected on a monthly basis and tested for anti‐SARS‐CoV‐2 antibodies against anti‐spike S1 domain. Thirty‐five hemodialysis patients were enrolled in the original study and 27 of these received a booster. Patients were followed up to 6 months after the first two doses and an additional 7 months after the third BNT162b2 dose. Results are presented as the internationally harmonized binding antibody units (BAU/mL).

**Results:**

Antibody level significantly increased from prebooster to 2 weeks postbooster, with a median [25th, 75th percentile] rise from 52.72 [28.55, 184.7] to 6216 [3806, 11,730] BAU/mL in the total population. Of patients with a negative or borderline detectable antibody level 6 months after vaccination who received a third dose, 89% developed positive antibody levels 2 weeks postbooster. Postbooster antibody levels declined an average rate of 29% per month in infection‐naïve patients. Antibody levels spiked in patients infected with SARS‐CoV‐2 after receiving a booster but declined rapidly. No patients infected postbooster required hospitalization.

**Conclusions:**

A third dose of BNT162b2 restores antibody levels to high levels in dialysis patients but levels decline over time. A third dose did not necessarily prevent infection, but no patients suffered severe infection or required hospitalization. SARS‐CoV‐2 recovered patients appear to have a blunted rise in antibody levels after a third dose. Although patients infected with SARS‐CoV‐2 postbooster had an immediate spike in antibody levels, these declined over time.

## 1 INTRODUCTION

In March of 2020, the World Health Organization (WHO) declared COVID‐19 a pandemic. Millions of deaths due to infection with this virus have occurred since this time. Containment strategies limiting the spread of the virus were a major focus early in the pandemic. Particularly worrying were patients who could not self‐isolate due to required hemodialysis sessions. Fortunately, vaccines against SARS‐CoV‐2 were rapidly developed, tested, and have been shown to be highly effective in the general population.^1^

Patients receiving hemodialysis are particularly vulnerable to infection with SARS‐CoV‐2. An increased incidence of SARS‐CoV‐2 infection has been reported in dialysis patients which may be in part explained by more frequent contact with health professionals and other high‐risk individuals.^2^, ^3^ Additionally, patients with CKD often have functional defects in innate and adaptive immunity and uremia related immune suppression.^4^ As a result, these patients may have an impaired ability to fight infection. Indeed, an increase in mortality to COVID‐19 in hemodialysis has been reported.^5^ Strategies to address this are critical not only to protect our hemodialysis patients but also to reduce the global burden of COVID‐19. Although hemodialysis patients obtain a robust antibody response immediately following a standard two‐dose vaccination series with Pfizer BioNTech (BNT162b2) mRNA SARS‐CoV‐2 vaccines, antibody levels wane over time.^6^, ^7^ Serological studies have also shown that hemodialysis patients obtain lower antibody responses to COVID‐19 vaccines, an unsurprising finding considering these patients have blunted responses to other vaccines including the Hepatitis B and pneumococcal vaccines.^8^, ^9^ Additionally, the two‐dose regimen is insufficient to protect patients from infection due to the Omicron variant.^10^ Boosters of SARS‐CoV‐2 mRNA‐based vaccines are recommended in dialysis patients and the general population.^11^ Reports have demonstrated high antibody levels following boosters in dialysis patients.^12^, ^13^, ^14^, ^15^, ^16^, ^17^, ^18^, ^19^ In one study, 91% of dialysis patients who received a third dose of BNT162b2 vaccine achieved antibody levels associated with protection, compared with only 35% prebooster.^12^ However, there are still few studies characterizing the long‐term humoral response postbooster in these patients.^16^, ^17^, ^18^, ^19^ Additionally, few studies directly compare the long‐term response after two doses of a COVID‐19 vaccine to the response after three doses in the same cohort of patients which can provide valuable insights regarding patient variability. Furthermore, studies in the general population have suggested that vaccinated individuals who have recovered from SARS‐CoV‐2 have high antibody levels.^20^ However, there remain few reports focused on this in dialysis‐specific populations. One study characterized the receptor binding domain‐specific memory B cell response in dialysis patients who were infected with SARS‐CoV‐2 before boosting.^21^ In the current study, we provide additional data characterizing the humoral response of dialysis patients. Additionally, we describe the humoral response in individuals who were infected after receiving a booster, a subgroup that has been understudied in hemodialysis patients.

Beginning in January of 2021, the University of Virginia began a dialysis program‐wide vaccination campaign using BNT162b2.^22^ We followed a cohort of these dialysis patients and reported their long‐term humoral responses to two doses of BNT162b2 for 6 months after full vaccination.^6^ A subset of these patients received a third dose of BNT162b2 and their short‐term antibody response was reported.^23^ Here, we complete the three‐part series to fully characterize the natural history of antibody levels in hemodialysis patients following mRNA‐based vaccine boosters. Specifically, we provide an additional 7 months of data supplementing our earlier descriptions of antibody levels targeting the receptor binding domain of the spike protein of SARS‐CoV‐2 following administration of a primary BNT162b2 vaccine series. Finally, we compare long‐term antibody levels after two doses with that after three doses.

## 2 MATERIALS AND METHODS

### 2.1 Study population

This study is an extension of an original study that originally enrolled 35 adults (>18 years) undergoing in‐center hemodialysis who were confirmed as fully vaccinated at the University of Virginia dialysis centers.^6^ Inclusion and exclusion criteria were previously reported.^6^ Patients requiring dialysis for acute kidney injury and those with active infection or requiring isolation for suspected SARS‐CoV‐2 infection were excluded from enrollment. The characteristics of these patients including age, gender, and comorbidities are outlined in Table 1 of our previous study.^6^ Of these 35 patients, 27 (77.1%) received a third dose of BNT162b2 and were followed for an additional 7 months. Over the course of the study, one patient withdrew consent, one received a kidney transplant, one was hospitalized for >30 days, and one switched to peritoneal dialysis. Six patients died from causes unrelated to SARS‐CoV‐2 infection. One patient was excluded since their booster vaccination date could not be confirmed.

**Table 1:** Descriptive summary of Nucleocapsid antibody level (presumed infection) status dynamically over time.

Timeline	Negative	Positive	Overall (*N* = 35)
May 2021	27 (81.2%)	6 (18.8%)	33
	178.8 [122.9, 409.9]	2097 [1298, 2499]	271.5 [139.6, 944.2]
June 2021	–	–	31
	–	–	252.8 [102.7, 699.9]
July 2021	–	–	32
	–	–	142.2 [68.89, 559.0]
August 2021	–	–	28
	–	–	101.9 [56.63, 298.3]
September 2021	–	–	28
	–	–	52.72 [28.55, 184.7]
*Booster*			
November 2021	20 (80%)	5 (20%)	25
	5986 [3680, 12,030]	6390 [3806, 8772]	6216 [3806, 11,730]
December 2021	18 (72.0%)	7 (28%)	25
	2566 [1660, 8539]	4877 [1574, 7483]	2654 [1650, 8340]
January 2022	18 (75%)	6 (25%)	24
	1444 [1092, 2080]	1458 [1344, 1883]	1444[1102, 2020]
February 2022	–	–	25
	–	–	2080[1463, 3682]
March 2022	11 (44%)	14 (56%)	25
	1818 [1247, 2319]	3629 [1687, 7383]	2281 [1449, 5040]
April 2022	12 (50%)	12 (50%)	24
	1204 [974.1, 1627]	2083 [1378, 5376]	1571 [1004, 2879]
May 2022	11 (50%)	11 (50%)	22
	879.5 [698.9, 1205]	1463 [78,648, 2109]	1103 [696.9, 1661]

*Note*: The antibody data are reported overall and separately by Nucleocapsid status over time and expressed as median (25th, 75th percentile) in BAU/mL.

### 2.2 Study setting

The University of Virginia (UVA) Health System operates 12 dialysis clinics throughout central Virginia. Peritoneal and home dialysis clinics are co‐located at select sites. In January 2021, the UVA Health System undertook a dialysis program‐wide vaccination effort in partnership with the Virginia Department of Health's Blue Ridge Health District (BRHD).^22^ UVA Health mobile vaccination clinics offered patients a two‐shot series of the Pfizer‐ BioNTech COVID‐19 vaccine (BNT162b2). Vaccinations were administered by dialysis pharmacists, nurses, and advanced practice nurses. Vaccine educational information was provided to patients on the day of vaccination. Consent or refusal for vaccination was obtained and documented.

Eligible hemodialysis patients from this vaccination campaign were recruited for the current study.

### 2.3 Sample collection and assessment

Samples from participants were obtained on a monthly basis beginning at an average of 9.1 weeks post full vaccination (defined as >14 days following second immunization) on designated collection dates for each dialysis shift (MWF or TTS). In October 2021, around 7 months after full vaccination, patients received a third dose of the BNT162b2 vaccine. Monthly samples were collected and processed beginning around 2 weeks (November 2022) following the third dose.

A 10 mL EDTA tube was collected from each patient's dialysis blood line during dialysis treatment, stored in a designated research refrigerator and processed within 8 h of initial collection. Tubes were centrifuged at 3000 rpm (1620rcf) for 10 min in the swing bucket rotor (S4180) at 4°C using a Beckman GS‐15R centrifuge. Plasma obtained was stored in −80°C in 0.5 mL aliquots until further analysis.

All monthly EDTA plasma samples were tested for anti‐SARS‐CoV‐2 antibodies against anti‐spike S1 domain using the commercially available Anti‐SARS‐CoV‐2 QuantiVac ELISA (IgG) from Euroimmun (EUROIMMUN US, Inc.). This test has high sensitivity (90.3%–93.2%) and specificity (99.8%).^24^ Samples above detection limits were re‐run with further dilution (1:5 or 1:10) in the sample buffer as recommended by the manufacturer. Based on the manufacturer's recommendation, final test results were presented in the internationally harmonized binding antibody units (BAU/mL). BAU/mL was obtained by multiplying the Relative Unit (RU/mL) by a factor of 3.2. Final test results were considered negative for BAU/mL (<25.6), borderline for BAU/mL (≥25.6 and <35.2), and positive for BAU/mL (≥35.2).^25^

The Bio‐Rad Platelia SARS‐CoV‐2 Total Ab assay (Bio‐Rad Laboratories, Inc.) was used for qualitative detection of total antibodies (IgM/IgG/IgA) to SARS‐CoV‐2 nucleocapsid protein to confirm prior infections and assess for unreported infections. Recombinant SARS nucleocapsid protein is used in the assay to capture total antibodies in a one‐step antigen capture format followed by detection.

### 2.4 Data collection

Demographic data including age, sex, race/ethnicity, and BMI and clinical data including comorbidities, use of immune suppressive medication, history of malignancy, and history of transplantation were obtained from the Electronic Health Record as previously described.^6^ Clinical information including dialysis vintage was obtained from the dialysis‐specific electronic medical record system. Prior COVID‐19 infection information was collected from a designated tracking file in the dialysis unit and verified with SARS‐CoV‐2 nucleocapsid protein assay results.

### 2.5 Statistical methods

Antibody levels were reported as medians (25th, 75th percentile) for their skewed distributions, and their differences over time (pre vs. post third dose) were compared using overall nonparametric repeated analysis of variance followed by post‐hoc test using Wilcoxon signed rank test. Bonferroni adjustment was applied for multiple comparison. The decline rate of antibody levels in the infection naïve subjects after booster was estimated from a linear mixed model with random intercept and random slope. Trend lines on graphs were approximated using loess method in ggplot2 package. *p* value < 0.05 was considered significant. All analyses were performed in R version 4.2.0.

## 3 RESULTS

**Figure 1:**
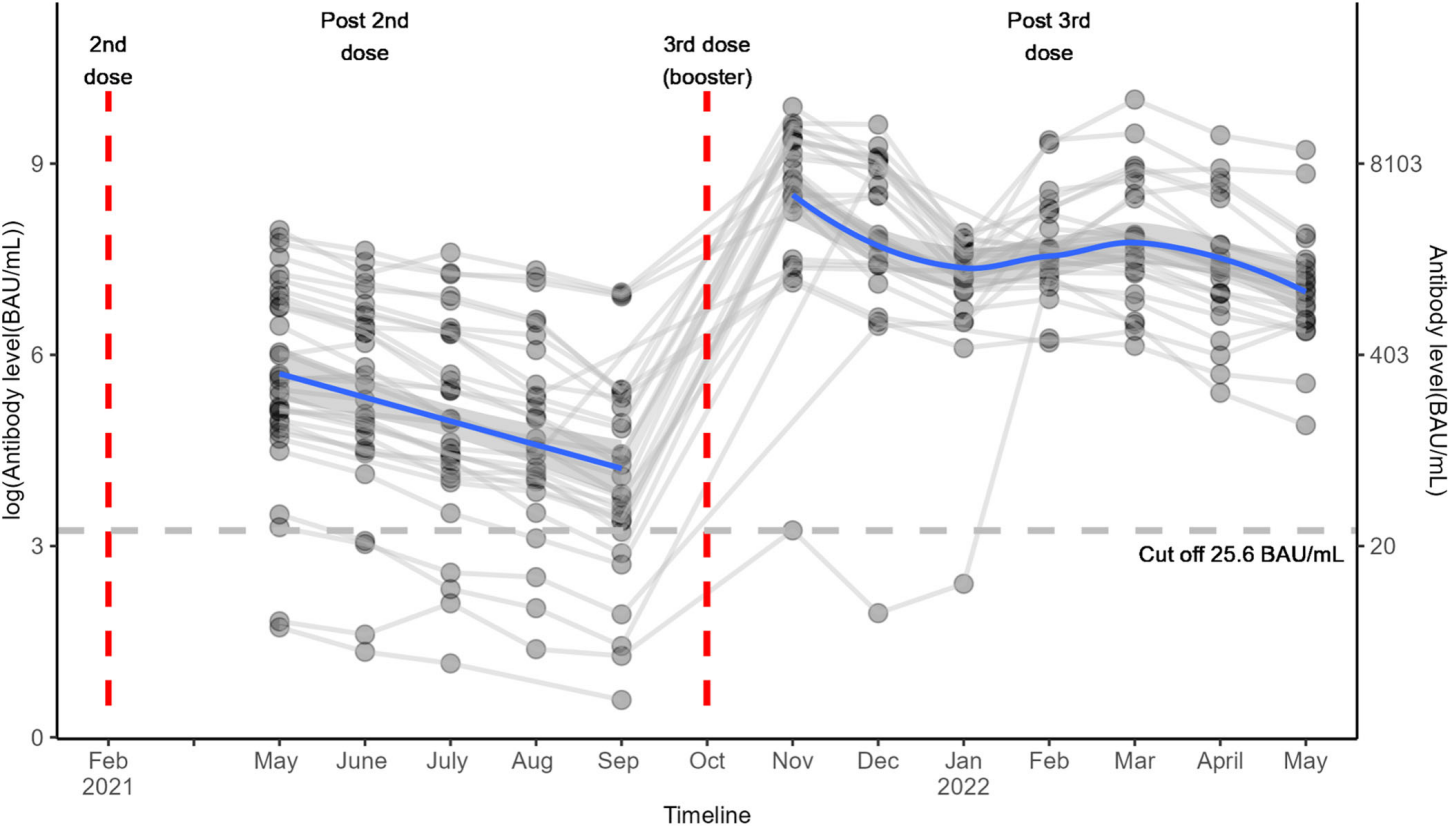
Trends in antibody level over time after SARS‐COV‐2 BNT162b2 vaccination. Each line represents a single patient trend. The horizontal dashed line is the positive/negative cutoff for antibody level protection (25.6 BAU/mL). The blue solid lines represent smoothed mean antibody level over time in pre‐ and postbooster periods.

**Figure 2:**
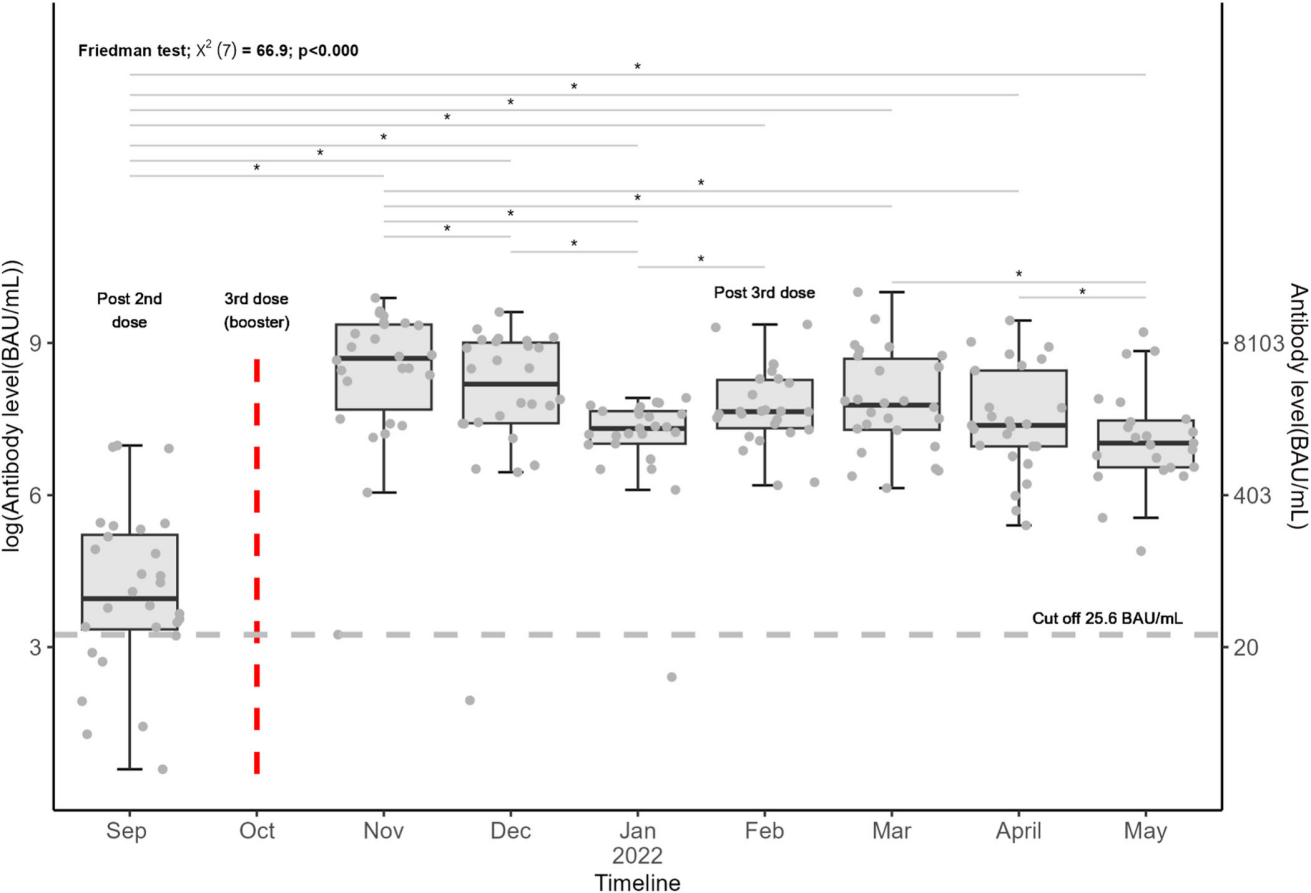
Boxplots of antibody level over time after a SARS‐COV‐2 BNT162b2 booster. Each dot represents a subject. The center line in each boxplot represents the median, and the lower and upper lines represent the 25th and 75th percentile, respectively. Friedman test was used for overall testing. “*” represents a statistically significant difference in antibody level between two‐time points. *p* value < 0.05 was considered significant. The horizontal dashed line is the positive/negative cutoff for antibody level protection (25.6 BAU/mL).

The patient characteristics in the study cohort (*N* = 35) were described in detail in our previous paper.^6^ Of these, 33 patients remained under observation as of May 2021, and 27 received a third dose of BNT162b2 (booster) in October 2021, with an additional 7 months of postbooster follow‐up. Temporal changes in antibody levels post two doses and post the third dose are presented in Table 1 and Figures 1 and 2. Details of the trajectories following two doses of the BNT162b2 vaccine were described previously.^6^ Unless specified otherwise, “prebooster” refers to the last time point before receiving the third dose (i.e., September 2021).

### 3.1 Long‐term humoral immunity trends

**Figure 3:**
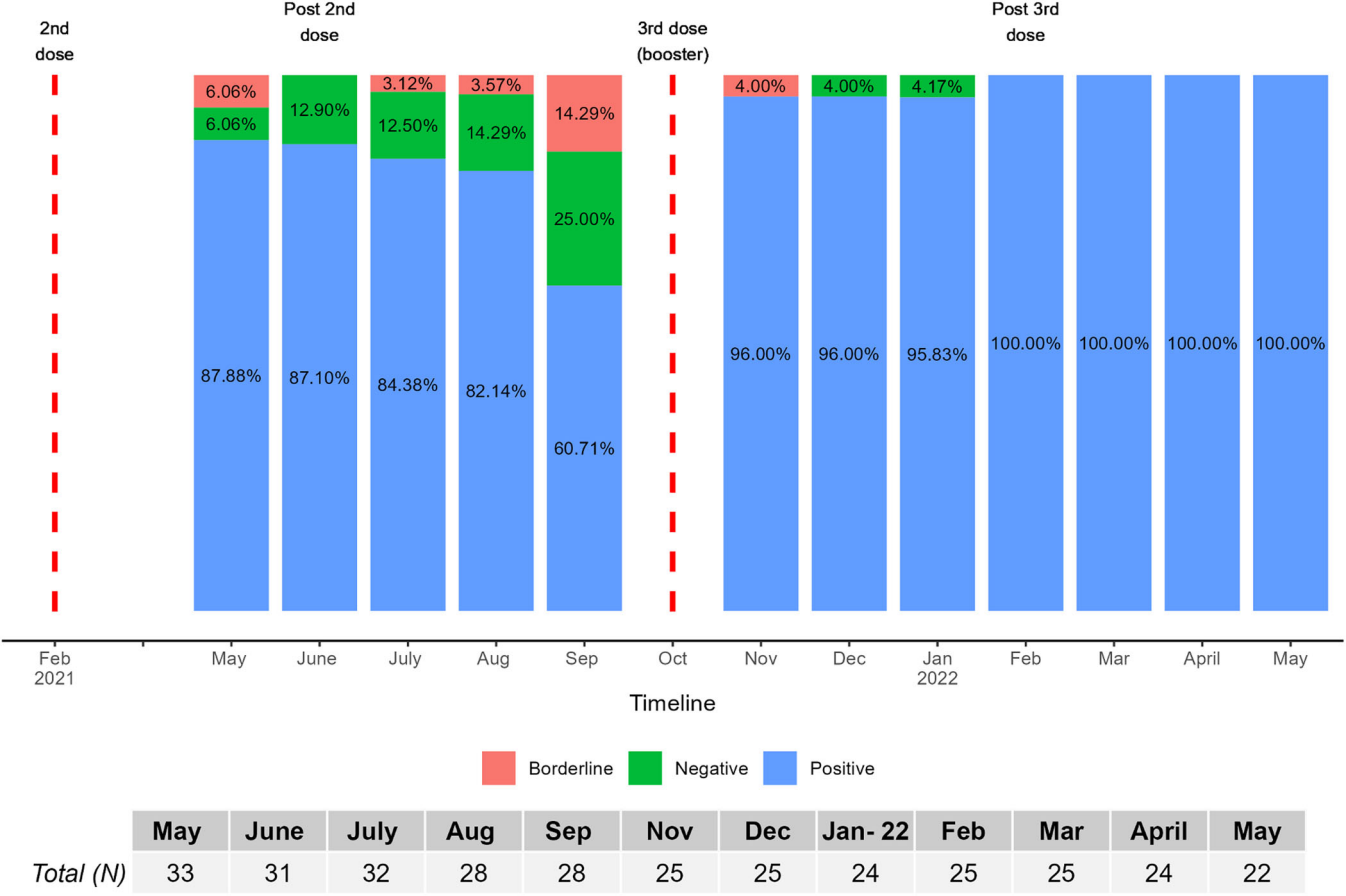
Percentage of subjects who were positive, negative, and borderline based on antibody level at each time during the study period. Percentages are calculated based on the total number of patient samples at each month. Final test results were considered negative for antibody <25.6 BAU/mL, borderline for 25.6 to <35.2, and positive for ≥35.2 BAU/mL.

Figure 1 shows that all subjects' antibody levels spiked following the third dose (i.e., Nov 2022). Friedman test showed antibody levels were significantly different over different time points (*p* < 0.0001, Figure 2), and all postbooster antibody levels were significantly greater than prebooster values with pairwise testing. Overall antibody level significantly increased from prebooster median [25th, 75th percentile]: 52.72 [28.55, 184.7] to 2 weeks postbooster (i.e., Nov 2021) 6216 [3806, 11,730] BAU/mL in the total population (Table 1 and Figure 1). In infection‐naïve patients, postbooster antibody levels declined at an average rate of 29% per month. Six months after the second dose, 61% of patients maintained positive antibody levels and 39% of the cohort had negative antibody levels. Conversely, 6 months after the booster dose, 100% of patients maintained positive antibody levels regardless of infection status (Figure 3).

Nine of the 11 patients with negative or borderline detectable antibody levels 6 months after primary series vaccination received a third dose of the BNT162b2 vaccine. Eight of these nine patients developed positive (≥35.2 BAU/mL) antibody levels 2 weeks postbooster (Figure 3). The one patient not achieving positive antibody levels also had negative antibody levels at the first sample collection following two BNT162b2 doses. Notably, this patient was immune suppressed. Six patients had prior SARS‐CoV‐2 infection as identified by a Bio‐Rad Platelia SARS‐CoV‐2 Total Ab assay assessing anti‐nucleocapsid IgG antibodies (Bio‐Rad Laboratories, Inc.). From prebooster to 2 weeks postbooster, those with prior infection had a lower proportional increase in antibody level (51 fold) compared with the median change in COVID naïve patients (144 fold).

### 3.2 Antibody levels post two or three doses

The median antibody level at first sample collection post 2 doses (~2 months post full vaccination, i.e., May 2021) was 178.8 [122.9, 409.9] BAU/mL in the infection naïve patients. Two months post the third dose, the median antibody level was 2566 [1660, 8539] BAU/mL in the infection naïve individuals, an average of 14‐fold higher. In all patients, at 6 months post the third dose, the median antibody level was 1571 [1004, 2879] BAU/mL, an average of 70‐fold higher compared with 6 months post two doses in the total population (Figure 2).

### 3.3 Infections post booster

**Figure 4:**
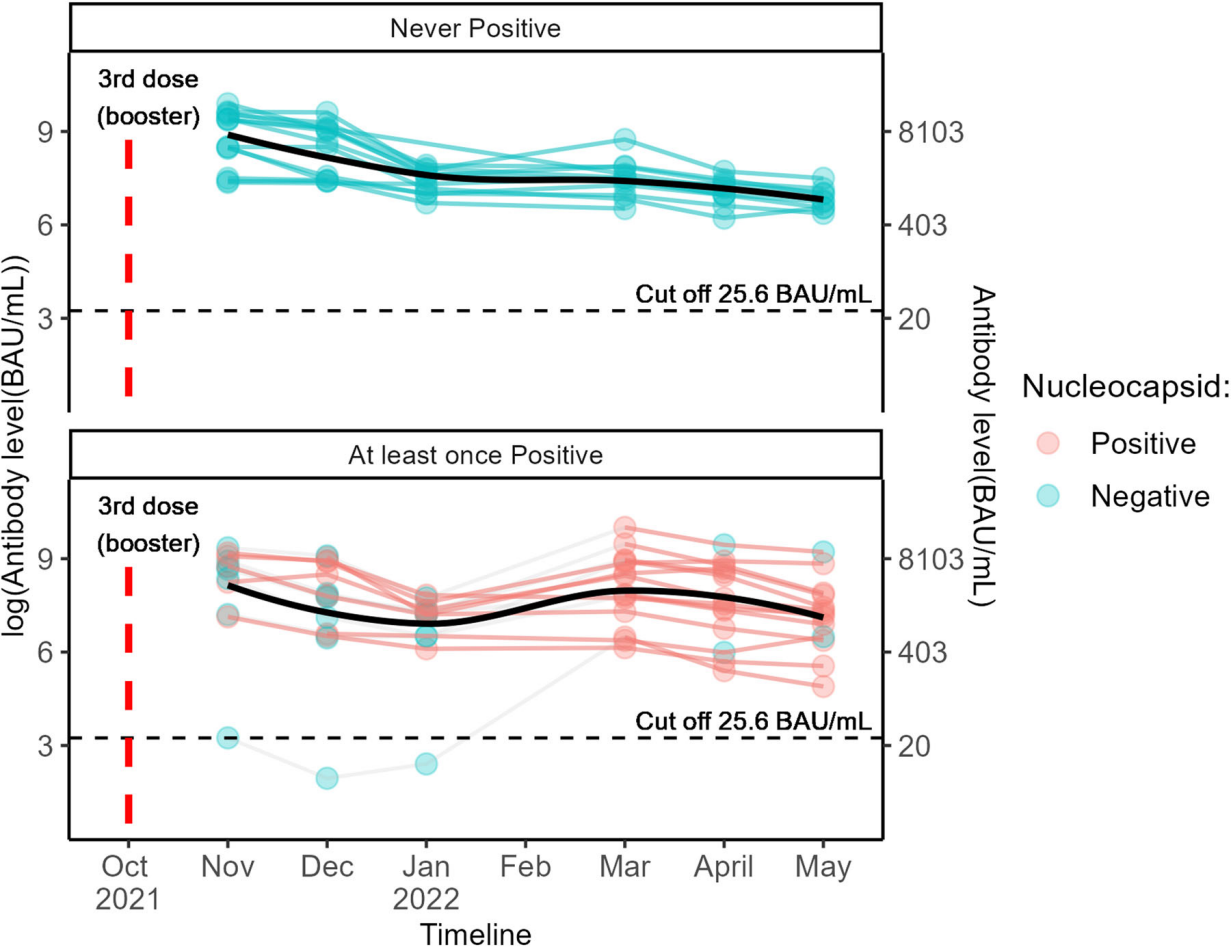
Antibody level over time after a SARS‐COV‐2 BNT162b2 booster, stratified by infection status over time. Each line represents a single patient trend. Color of each dot represents nucleocapsid status for that month. Subjects in the top never had an infection during the study period as determined by nucleocapsid result, while those in the bottom had at least one positive nucleocapsid result. The horizontal dashed lines are the positive/negative cutoff for antibody level protection (25.6 BAU/mL). The black solid lines represent smoothed mean antibody level over time.

The Bio‐Rad Platelia SARS‐CoV‐2 Total Ab assay identified an additional nine patients (33%) who became infected postbooster. Only one of these patients had a diagnosed infection in the medical record. None of these patients required hospitalization for treatment. A significant portion of these patients (eight patients, 89%) had a positive nucleocapsid beginning March of 2022 (Figure 4) which corresponds to the period of circulating Omicron variant.

## 4 DISCUSSION

Despite clear evidence supporting the vulnerability of dialysis patients to infection with SARS‐CoV‐2, there are relatively few reports focused on vaccine response specifically in this population especially considering the number of patients receiving dialysis worldwide. Studies have shown that, although a majority of dialysis patients attain positive antibody levels following a standard two‐dose vaccine regimen, these responses are lower than that of the general population. Particularly scarce are studies following the same cohort of dialysis patients after two and three doses of a SARS‐CoV‐2 vaccine. Here, we characterized the trajectories of antibody levels 6 months after two vaccine doses and 7 months after three doses in the same population. After excluding patients who were infected with COVID‐19 during the study period, antibody levels 2 months post three doses were significantly higher compared with 2 months post two doses. Although antibody levels after receiving a booster declined over time, at 6 months post three doses, antibody levels remained significantly higher than 6 months post two doses regardless of infection status. Thus, the three‐dose regimen is effective in both the short and long‐term in raising antibody levels compared with the two‐dose regimen. Similar trends of antibody decline over time are observed in healthy individuals, although dialysis patients may differ from the general population with reduced peak levels and lower seroconversion rates.^26^ Long‐term durability remains unclear and protective levels against infection are unknown. Goldblatt et al. reported the mean protective threshold against WT SARS‐CoV‐2 virus was 154 BAU/ml (95% CI 42–559) but higher levels are presumed required against newer variants.^27^

In the general population, it has been suggested that vaccination plus occult infection provides high antibody levels.^20^, ^28^ It is unclear whether this holds true in dialysis cohorts. Thus far, there is only one report focused on this in dialysis‐specific populations.^21^ In this study, the investigators characterized the memory B cell response in virus‐naïve and SARS‐CoV‐2 recovered patients on dialysis after boosting. In their cohort of 39 boosted dialysis patients, 13 were previously infected with SARS‐CoV‐2 (with 26 virus‐naïve patients). In these patients, antireceptor binding domain memory B cells remained unchanged after the third dose. In our cohort, previously infected patients saw a blunted rise in antibody levels after a third dose compared to their infection naïve counterparts, though these previously infected patients started from a higher baseline. Thus, overall, they attained similar peak levels. The report by Attias et al. focused on patients infected before receiving a booster dose. However, the antibody response of hemodialysis patients who became infected after boosting has been less well described. While patients in our study who were infected postbooster had an immediate spike from what had previously been declining antibody levels, these values declined rapidly in subsequent months. This suggests that a SARS‐CoV‐2 infection pre‐ or postbooster may not necessarily result in higher long‐term antibody levels, although longer‐term follow‐up postinfection is needed. Additionally, if humoral immunity is critical in disease prevention or mitigation, additional doses may be required in these patients, even after a third dose plus infection.

We recently reported the initial results of antibody levels in these patients up to 11 weeks after boosting.^23^ Interestingly, immediately following this, we observed a spike in antibody levels in a large proportion of our patients. A significant number of patients developed a positive nucleocapsid in March of 2022, which coincided with the B.1.1.529 (Omicron) variant wave (nucleocapsid results were not obtained for the month of February). Notably, only one of the newly nucleocapsid positive patients had a documented record of infection. This has multiple important implications. First, it suggests an underreporting of true infection rates even in patients in constant contact with the health system subject to pretreatment screenings. Of note, the pattern of infected patients was not consistent with intrafacility transmission, though this cannot be completely ruled out. Additionally, while boosting/high antibody levels was not effective in preventing infection in all individuals, no patients experienced hospitalization or severe illness. This is in line with a recent report of 1126 hemodialysis patients, 437 of which received a third dose of either BNT162b2 or mRNA‐1273 (Moderna).^29^ While a third dose did not eliminate infection compared with two doses, it may have conferred additional protection from severe infection. This is important in a population that has been shown to experience more severe COVID‐19 outcomes compared with nonkidney disease patients.^30^

Notably, Wang et al. compared the neutralizing antibody response in dialysis patients to both WT and omicron variants and found that the vaccine response against Omicron was significantly less compared with that against WT.^31^ Additionally, the half‐life of the omicron antibody response was shorter than that of the WT. These findings likely account for our rapid increase in antibody level in patients who were infected postbooster and the subsequent rapid decline. While we did not perform SARS‐CoV‐2 genomic sequencing, based on local surveillance at the time, it is assumed that most of these patients were infected with the Omicron variant.

Our study has limitations worth noting. First, we did not perform variant‐specific neutralization assays and thus we cannot determine booster effectiveness against different variants.^14^ Nor did we assess cellular immunity responses in this study. Longer‐term follow‐up after patients developed a SARS‐CoV‐2 infection is needed to fully characterize the humoral response in this group of individuals. Our relatively small cohort precludes deeper analysis into the role of comorbidities in vaccine response. Since all of our patients continuing in the study past 6 months opted to receive a third vaccine dose, we are unable to compare outcomes to patients who received only two vaccine doses. Finally, we report on antibody response solely to BNT162b2 and not other COVID‐19 vaccines, although this may also be considered as a strength.

## 5 CONCLUSION

In conclusion, we demonstrate that antibody levels decline in hemodialysis patients after two doses of BNT162b2 but a third dose greatly, but transiently, increases antibody levels to high levels. SARS‐CoV‐2 recovered patients have a blunted rise in antibody levels after a third dose, but start at a higher baseline, thus attaining similar peak levels compared to their infection‐naïve counterparts. Patients infected with SARS‐CoV‐2 postbooster have an immediate spike in antibody levels, however these similarly decline over time. A booster dose does not preclude SARS‐CoV‐2 infection in hemodialysis patients but may confer protection against severe infections leading to hospitalization or death. These findings underscore the importance of administering additional vaccine doses in hemodialysis patients even in boosted and SARS‐CoV‐2 recovered patients.

## AUTHOR CONTRIBUTIONS

**Eibhlin Goggins**: Conceptualization; investigation; methodology; project administration; supervision; validation; visualization; writing—original draft; writing—review and editing. **Binu Sharma**: Formal analysis; software; validation; visualization; writing—original draft; writing—review and editing. **Jennie Z. Ma**: Formal analysis; validation; visualization; writing—review and editing. **Jitendra Gautam**: Data curation; methodology; resources; validation; writing—original draft; writing—review and editing. **Brendan Bowman**: Conceptualization; funding acquisition; investigation; methodology; project administration; resources; supervision; validation; writing—original draft; writing—review and editing.

## CONFLICT OF INTEREST STATEMENT

The authors declare no conflict of interest.

## ETHICS STATEMENT

This research proposal was reviewed and approved by the University of Virginia Institutional Review Board for Health Sciences Research (tracking number: HSR 210095).

## TRANSPARENCY STATEMENT

The lead author Eibhlin Goggins affirms that this manuscript is an honest, accurate, and transparent account of the study being reported; that no important aspects of the study have been omitted; and that any discrepancies from the study as planned (and, if relevant, registered) have been explained.

## Data Availability

The data set used for this analysis is not publicly available. The data utilized was obtained from the Electronic Health Record and from the dialysis‐specific electronic medical record system at the University of Virginia Health System which is restricted to use by only authorized employees.
